# INCREASED INCIDENCE OF TUBERCULOSIS IN PATIENTS OF SYSTEMIC SCLEROSIS ON DEXAMETHASONE PULSE THERAPY: A SHORT COMMUNICATION FROM KASHMIR

**DOI:** 10.4103/0019-5154.39737

**Published:** 2008

**Authors:** Qazi Masood Ahmad, Iffat Hassan Shah, Qazi Nauman, Farah Sameem, Javaid Sultan

**Affiliations:** *From Department of Dermatology, STD and Leprosy, Government Medical College, Srinagar, Kashmir, Jammu and Kashmir, India*

**Keywords:** *Dexamethasone pulse*, *systemic sclerosis*, *tuberculosis*

## Abstract

**Background:**

Systemic sclerosis is a multi-systemic autoimmune disorder affecting predominantly the skin, lungs, gut and kidneys.

**Purpose:**

To report the increased incidence of tuberculosis in patients of systemic sclerosis on dexamethasone pulse (DP) therapy.

**Methods:**

Forty-seven patients of systemic sclerosis were included in the study. After taking a complete history and doing a detailed physical examination, the patients were submitted to a battery of investigations including complete hemogram (CBC) with erythrocyte sedimentation rate (ESR(F)), Chest X-ray CXR (PA view) Mantoux test and urine analysis. CBC, ESR and urine examination was done monthly and CXR were repeated six-monthly.

**Findings:**

Seven patients on DP therapy developed genitourinary tuberculosis. Four had pulmonary tuberculosis. One patient developed tubercular lymphadenitis, one patient succumbed to miliary tuberculosis.

**Conclusion:**

There is an increased incidence of tuberculosis amongst patients of systemic sclerosis on DP therapy.

**Limitation of the Study:**

There was no control group of systemic sclerosis patients not on DP therapy to rule out the confounding effect of the disease per se predisposing to tuberculosis as all our patients as a matter of routine were put on steroid pulse. Also, the increased incidence of tuberculosis was detected incidentally while on monthly follow-up.

## Introduction

Systemic sclerosis is a multi-systemic autoimmune disorder affecting predominantly the skin, lungs, gut and kidneys. The criteria to be fulfilled for labeling a patient as a case of systemic sclerosis (established by the Sub-committee for Scleroderma Criteria of the American Rheumatology Association (ARA)) includes one major criterion i.e. sclerosis of skin proximal to the digits including the face, limbs, neck or trunk and/or two or more minor criteria 1) Sclerodactyly 2) Digital pitted scarring 3) Bilateral Lower zone (basal) pulmonary fibrosis.^1^

Steroids in the form of intravenous dexamethasone pulse (DP) therapy have been used in the Department of Dermatology STD and leprosy, SMHS (Associated teaching hospital of GMC Srinagar) since 1999.

## Aim of the Study

To report our experience regarding the increased incidence of tuberculosis in patients of systemic sclerosis on DP therapy.

## Methods

Forty-seven patients of systemic sclerosis admitted in the in-patient wing of the Department Of Dermatology, STD and Leprosy SMHS Hospital Srinagar during 1999-2005 were included in the study. The diagnosis of systemic sclerosis was made on the basis of the ARA criteria. A complete history including any past history of tuberculosis was taken. A detailed physical examination including palpation for any lymphadenopathy and auscultation for chest signs was done. A meticulous cutaneous examination was done. The patients were submitted to a battery of investigations including complete hemogram (CBC) with erythrocyte sedimentation rate (ESR), Chest X-ray (CXR) PA view, Mantoux test and urine analysis. All these investigations were done at baseline. CBC, ESR and urine examination was done monthly and CXR were repeated six-monthly. Patients with positive Mantoux were put on INH prophylaxis.

After the pretreatment assessment patients were put on intravenous infusions of 100 mg dexamethasone in 500 ml of D5% on three consecutive days per month for 18-24 months or till patients showed no further improvement over further three months of therapy.

## Results

Forty-seven patients of systemic sclerosis were included in the study. They included 45 females and two males (F:M ratio of 23:1). The age range of patients was 18-60 years (average age of presentation was 40 years). The duration of disease prior to presentation ranged from one month to 25 years. Fifteen patients were grossly underweight for height. Two patients gave a previous history of pulmonary tuberculosis with ATT intake (>3 years before). The patients were put on DP therapy (only 43 patients, as those with Mixed Connective Tissue Disease were excluded from the study).

Seven patients on DP therapy on monthly follow-up had persistent asymptomatic pyuria. Their urine was sent for routine bacterial culture. This was negative. Culture for acid fast bacilli (AFB) was done. ESR (F) and Polymerase chain reaction (PCR) of overnight urinary sediment for Mycobacteruim tuberculosis was then sent to exclude genitourinary tuberculosis. Of these, five patients had PCR +ve for Mycobacterium tuberculosis. Most of these patients had taken 8-12 DPs. Four patients had chest symptoms in the form of dry cough/cough with expectoration. Chest auscultation revealed crepitations. Sputum was sent for AFB on three consecutive days and CXR (PA view) was repeated. Broncho-alveolar lavage (BAL) with AFB staining and PCR of lavage fluid was done. All four had BAL-confirmed pulmonary tuberculosis. These patients had taken longer therapy (24 pulses). One patient developed FNAC proven tubercular lymphadenitis (cervical) after 3 DPs. One patient succumbed to miliary tuberculosis after three DP's.

## Discussion

Steroid pulse therapy has been used in many immune-mediated disorders like pemphigus, bullous pemphigoid, systemic lupus erythmatosus, rheumatoid arthritis and pyoderma gangrenosum.

Pai, Srinivas, Sabetha *et al.* mention reference used DP therapy in systemic sclerosis but unfortunately only in too few a number of patients. They reported some beneficial effects of steroid pulse therapy in systemic sclerosis.^2^

Masood, Hassan, Majid^3^ have reported the preliminary results of DP therapy in systemic sclerosis. In their study of 10 patients completing therapy, there was one case of tubercular cervical lymphadenopathy.

## Conclusion

In view of the fact that in our study four patients developed pulmonary tuberculosis and five patients developed genitourinary tuberculosis after therapy the beneficial effects of DP therapy need to be reviewed vis-á-vis the side-effects.

It may be mentioned here that one patient who had positive Mantoux prior to therapy and was put on INH prophylaxis developed pulmonary tuberculosis after 24 DPs. Interestingly, no case of genitourinary or pulmonary tuberculosis developed in any patient of pemphigus (67) receiving dexamethasone cyclophosphamide pulse therapy in a parallel ongoing study in our Department. This despite the fact that patients in the two groups had a similar demographic profile and that they were receiving cyclophosphamide daily and in pulse form in addition to dexamethasone alone. This leads one to conjecture that systemic sclerosis per se may be responsible for this finding of an increased incidence of tuberculosis after DP therapy. Mantoux test and CXR (PA view) is hence done in all patients of systemic sclerosis in our wards now and immuno suppressants are preferably withheld in Mantoux positive patients.
